# Post-surgery survival and associated factors for cardiac patients in Ethiopia: applications of machine learning, semi-parametric and parametric modelling

**DOI:** 10.1186/s12911-024-02480-9

**Published:** 2024-03-29

**Authors:** Melaku Tadege, Awoke Seyoum Tegegne, Zelalem G. Dessie

**Affiliations:** 1 College of Science, Bahir Dar University, Bahir Dar, Ethiopia; 2 Department of Statistics, Injibara University, Injibara, Amhara, Ethiopia; 3 Regional Data Management Center for Health (RDMC), Amhara Public Health Institute (APHI), Bahir Dar, Ethiopia; 4 School of Mathematics, Statistics and Computer Science, University of KwaZulu- Natal, Durban, South Africa

**Keywords:** Cardiac disease patients, Machine learning, Parametric regression, Survival, Log-rank test, Cardiac surgery, Ethiopia

## Abstract

**Introduction:**

Living in poverty, especially in low-income countries, are more affected by cardiovascular disease. Unlike the developed countries, it remains a significant cause of preventable heart disease in the Sub-Saharan region, including Ethiopia. According to the Ethiopian Ministry of Health statement, around 40,000 cardiac patients have been waiting for surgery in Ethiopia since September 2020. There is insufficient information about long-term cardiac patients’ post-survival after cardiac surgery in Ethiopia. Therefore, the main objective of the current study was to determine the long-term post-cardiac surgery patients’ survival status in Ethiopia.

**Methods:**

All patients attended from 2012 to 2023 throughout the country were included in the current study. The total number of participants was 1520 heart disease patients. The data collection procedure was conducted from February 2022- January 2023. Machine learning algorithms were applied. Gompertz regression was used also for the multivariable analysis report.

**Results:**

From possible machine learning models, random survival forest were preferred. It emphasizes, the most important variable for clinical prediction was SPO2, Age, time to surgery waiting time, and creatinine value and it accounts, 42.55%, 25.17%,11.82%, and 12.19% respectively. From the Gompertz regression, lower saturated oxygen, higher age, lower ejection fraction, short period of cardiac center stays after surgery, prolonged waiting time to surgery, and creating value were statistically significant predictors of death outcome for post-cardiac surgery patients’ survival in Ethiopia.

**Conclusion:**

Some of the risk factors for the death of post-cardiac surgery patients are identified in the current investigation. Particular attention should be given to patients with prolonged waiting times and aged patients. Since there were only two fully active cardiac centers in Ethiopia it is far from an adequate number of centers for more than 120 million population, therefore, the study highly recommended to increase the number of cardiac centers that serve as cardiac surgery in Ethiopia.

**Supplementary Information:**

The online version contains supplementary material available at 10.1186/s12911-024-02480-9.

## Introduction

CVD was responsible for approximately 19.1 million deaths worldwide in 2020. Sub-Saharan Africa had the highest CVD-related mortality rates in 2020. It was estimated that 244.1 million people worldwide would live with ischemic heart disease alone in 2020, not including other heart diseases [1]. In the United States, one person dies from cardiovascular disease every 33 s [2].

Cardiovascular diseases are the number one cause of death [3]. Approximately one-third of CVD patients will require surgical or interventional care at some point [4]. However, about 6 billion people worldwide, primarily in low- and middle-income countries, lack access to cardiac surgery [5]. Congenital heart disease, rheumatic heart disease, and ischemic heart disease account for the lion’s share of the global CVD burden that necessitates surgical intervention [6].

Even though cardiovascular disease is the primary cause of mortality and morbidity, approximately, about 6 billion people on the globe lack access to timely, safe, and affordable cardiac surgery [7]. In low and middle-income countries, access to such services is also disproportionately low [4].

Furthermore, the risk factors for non-communicable diseases are increasing unacceptably rapidly in low- and middle-income countries, including Africa [8–10]. Chronic diseases, including CVD, are rising in low- and middle-income countries, posing a public health crisis [11, 12]. Aside from the increased burden of CVDs in LMICs, there is a scarcity of data on CVD risk factors [12].

Africa faces several challenges due to its CVD burden, including a lack of high-quality data, competing priorities, financial constraints, diagnostic, limited skill sets, and management challenges [13]. Africa has over one billion people and contributes significantly to the global CVD burden [14]. In 2013, CVD was responsible for an estimated one million deaths in Sub-Saharan Africa alone, accounting for 5.5% of all global CVD-related fatalities and 11.3% of all deaths in Africa [15]. In 2019, CVD was responsible for over one million deaths in Sub-Saharan Africa alone [16].

Estimating the CVD burden in Sub-Saharan African countries, including Ethiopia, is difficult [17]. According to the World Health Organization, around 30% of Ethiopians died from non-communicable diseases in 2014, with CVD accounting for 9% [18]. According to a systematic review conducted in Ethiopia, the prevalence of CVD ranges from 7.2 to 24% [19].

Due to a lack of workforce, infrastructure, and resources, Ethiopia has limited cardiac procedures, with long wait lists [6]. Ethiopia is a low-income country in East Africa, with approximately 120 million people [20]. Low-income countries are estimated to require 300 to 400 cardiac operations per million yearly population [7]. Despite the high demand for cardiac interventions, Sub-Saharan Africa has a scarcity of such services [21, 22]. There is still a scarcity of literature on cardiac surgery in Ethiopia [6].

According to recent studies, the elderly population over 60 is expected to grow from 962.3 million to 2080.5 million between 2017 and 2050 [23]. The heart-related figure is not known in developing countries [24]. There is little data on heart disease prevalence, especially in developing countries [25–27]. In recent years, the Ethiopian population has seen a significant epidemiological shift. The primary causes of illness and death change from infectious to noncommunicable diseases [28]. There is a deficiency in developing and executing novel solutions to lower the risks associated with cardiovascular illnesses because there is a mistaken perception that cardiovascular disease is solely a problem of the prosperous and industrialized world. There is also a tendency to associate heart disease mainly with a sedentary lifestyle and hyper-nutrition [29, 30]. Heart disease is neglected by health economists and experts [31].

The report from Cardiac Center Ethiopia shows that the number of cardiac disease patients increases dramatically from time to time; this is why many patients require cardiac surgery. According to the Ministry of Health statement, around 40,000 cardiac patients have been waiting for surgery in Ethiopia since September 2020. According to the Cardiac Center Ethiopia report, more than 7000 patients seek surgery at Ethiopia’s cardiac center [32].

Limited information about the current burden status of cardiac disease in Ethiopia is available. Therefore, this study provided an up-to-date assessment to evaluate the cardiac burden after surgery. There is no sufficient report on cardiac disease, specifically on the survival status of patients after surgery in Ethiopia. The objective was to determine the main predictors of post cardiac surgery survival in Ethiopia.

## Method and participants

### Study area and period

Ethiopia is a nation in the horn of Africa. It is bounded to the north by Eritrea and Djibouti, to the northeast by Somaliland, to the east by Somalia, to the south by Kenya, to the west by South Sudan, and the northwest by Sudan. Ethiopia has more than 120 million people, making it the 12th most populated country globally and the second most populous in Africa.

**Fig. 1:**
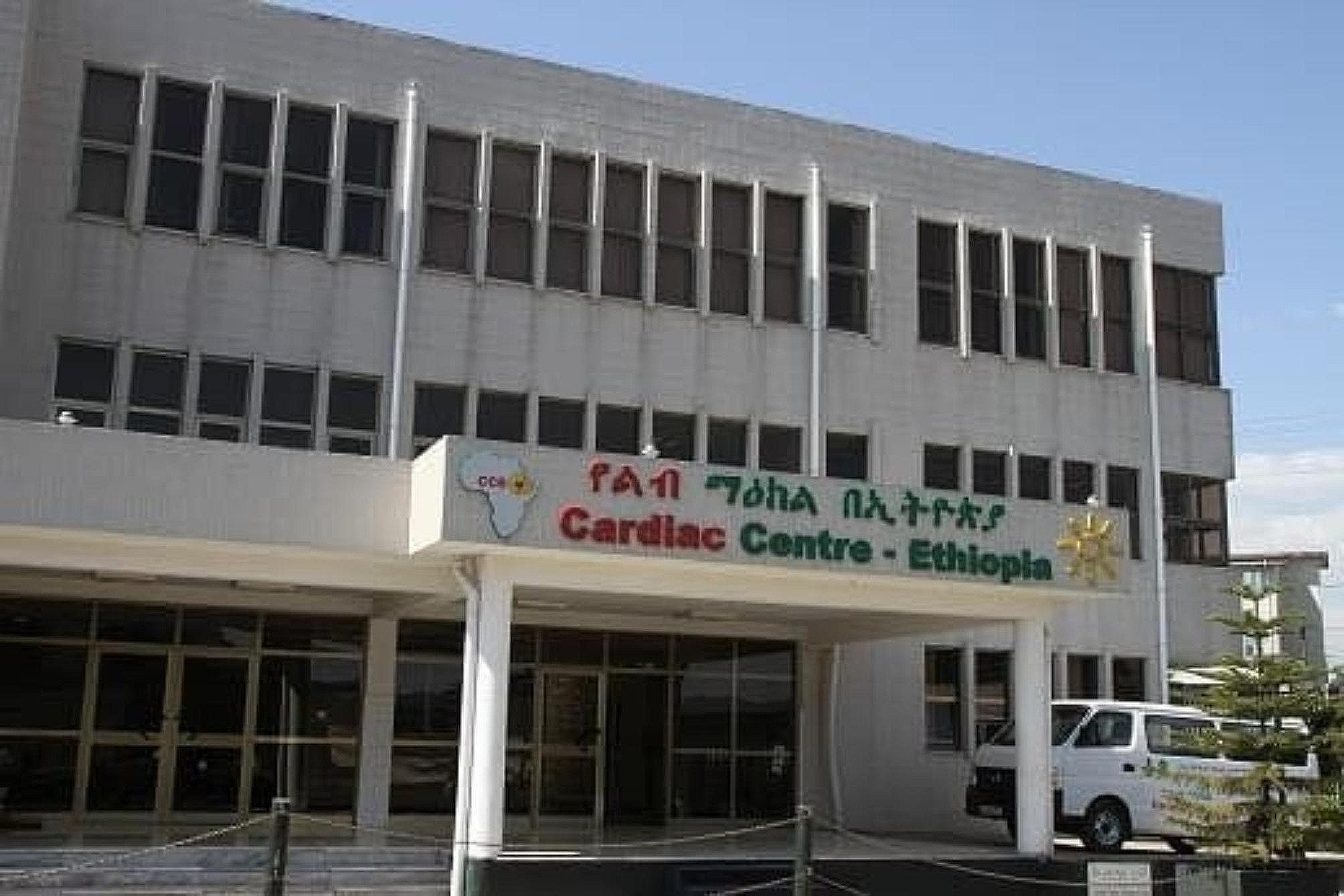
Cardiac center Ethiopia

The government has made enormous expenditures in the public health sector, which has resulted in improved health outcomes. Nonetheless, the Ethiopian government commitment or tangible effort to establish fully functional cardiac centers is limited. This study was focused on cardiac surgery patients and those registered for follow-up treatments in Ethiopia. The study was conducted at Cardiac Centre Ethiopia and Elouzeir Cardiac Center, which actively provided cardiac surgery during the study period. A non-governmental organization owns Cardiac Center Ethiopia, Children Heart Fund Ethiopia, and currently provides Ethiopia’s most significant cardiac surgery. The center started to provide surgical management for cardiac patients in 2009. The study was conducted on the patients’ follow-up charts from 2012 to 2023. The following figure shows one of the study areas called cardiac center Ethiopia (Fig. 1).

### Study design

The study was carried out through a retrospective cohort study design. The source of the population was cardiac patients whose follow-ups were between 2012 and 2023 in Ethiopia.

#### Study population

The study population was heart patients who had cardiac surgery between 2012 and 2023 in Ethiopia. Two cardiac centers in Ethiopia serve as cardiac surgery. All cardiac centers were part of the study. In the study area, 1520 patients had cardiac surgery. All 1520 patients were included in the study.

#### Data collection technique

The data were collected using structured questionnaires which was developed by the study (see Supplementary file 1) through an online data collection platform (Kobo tool box). BSc nurses and medical doctors participated in the data collection process. Data collection started after ethical clearance was obtained from Bahir Dar University. The permission was obtained from health provider institutions.

#### Data quality assurance method

The questionnaire was evaluated in randomly selected charts and participants; as a result, the pre-test was conducted to check data quality. Trained health professionals performed the data collection. Data consistency was supervised and reviewed by the investigator every day.

### Variable under the study

**Fig. 2:**
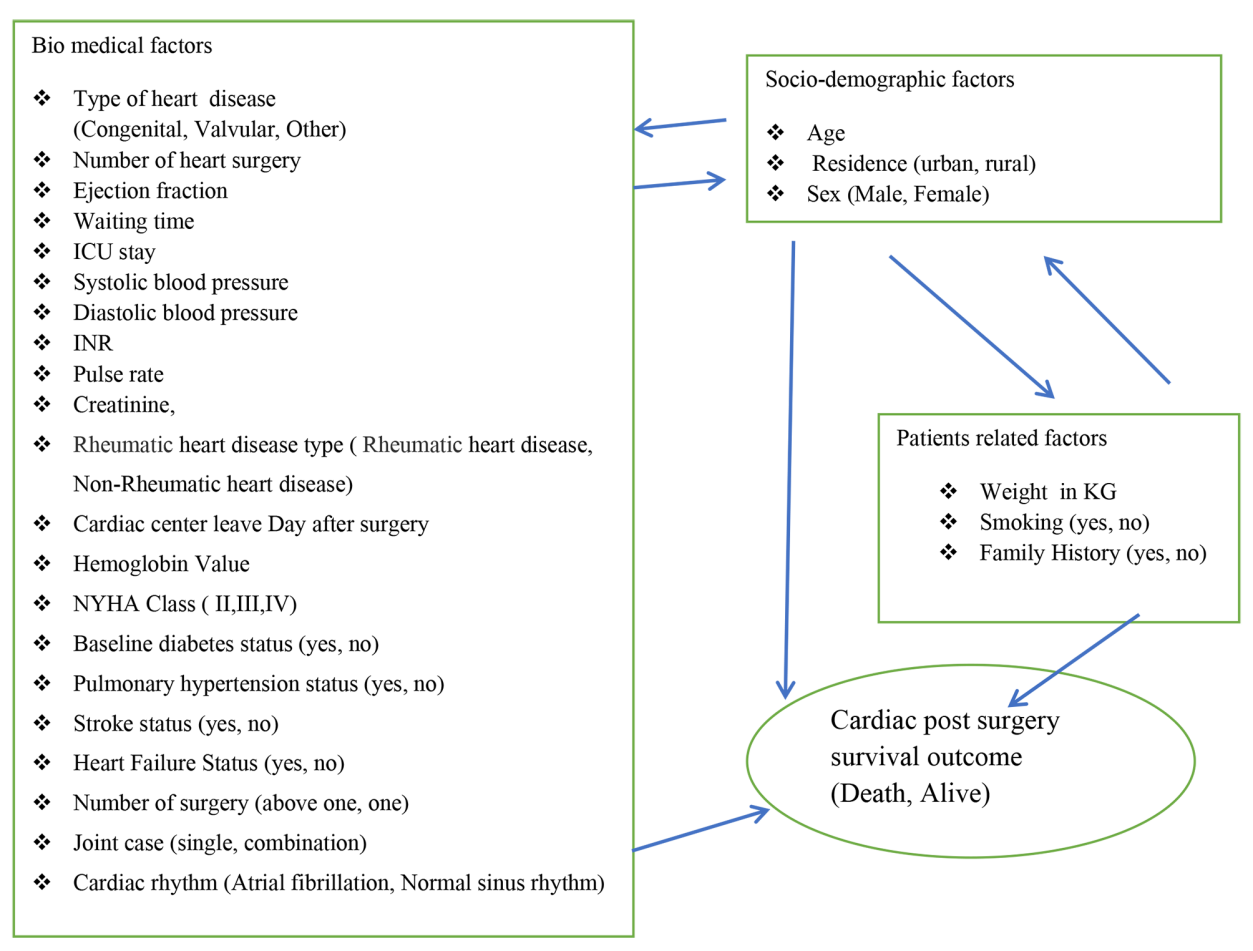
Predictor variables framework

Outcome variable: The outcome variable for survival analysis (Alive/censored, Death) outcome. The predictor variables: - The predictor variables are summarized as indicated in (Fig. 2).

### Data processing, importance variable selection and analysis techniques

**Fig. 3:**
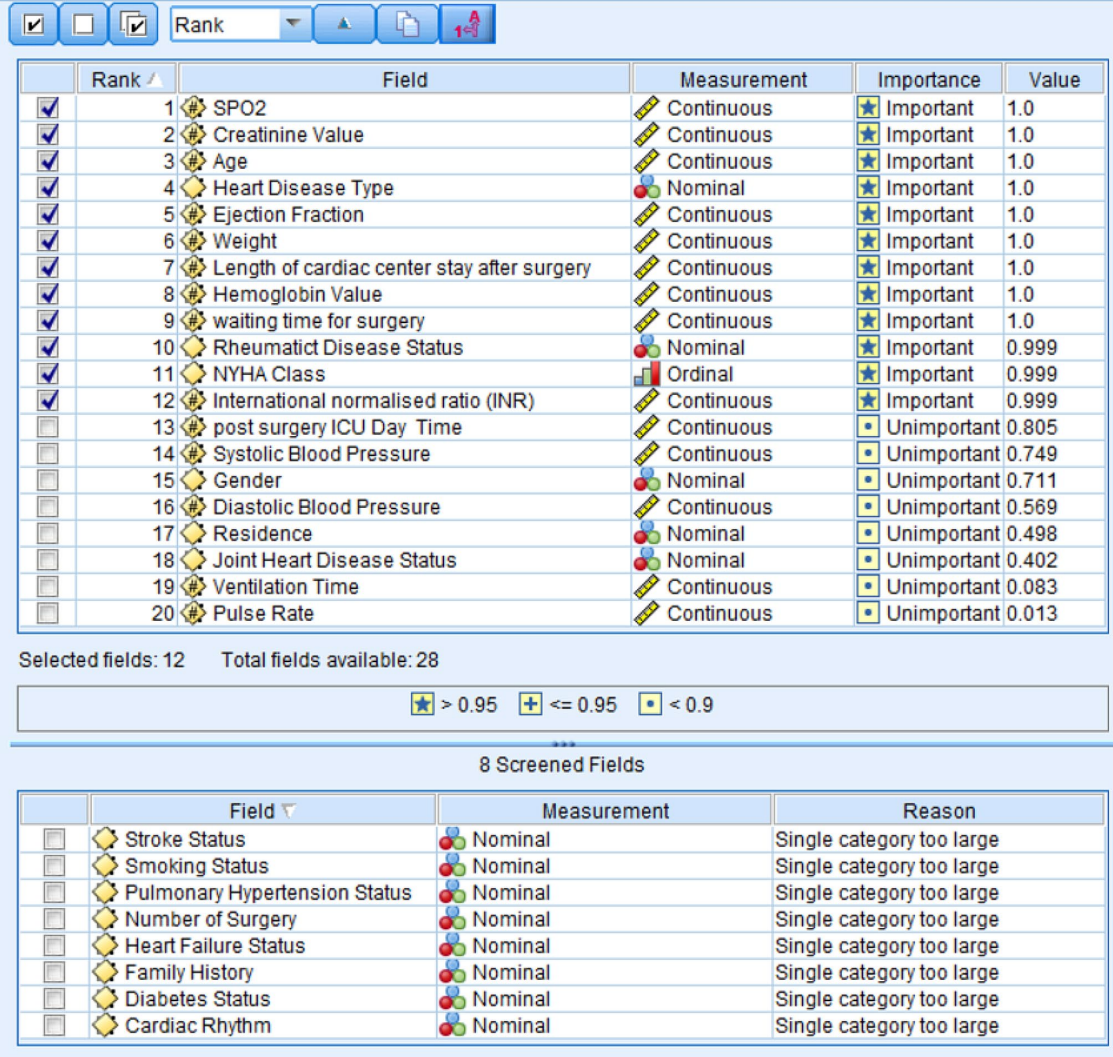
Important variable extraction output

After data collection, the collected data exported in to excel. Descriptive statistics characterise the study population in terms of relevant variables. The descriptive result is stated in the form of tables. Test of the association performed using chi-square test of association. Survival mean time comparison was done using t test. Different machine learning algorithms were considered. Feature selection, auto data preparation, and analysis were performed using IBM SPSS Modeler software. Python and Stata version 13 was used also for survival machine learning, semi parametric and parametric regression model comparison, analysis and model adequacy. From all 28 variables, 12 were selected using the machine learning variable selection method. The study used a feature selection procedure, ranking the importance of inputs relative to a specified target. Among all predictor variables, 12 variables were included in the model (Fig. 3).

### Machine learning for survival outcome

The most important feature of machine learning algorithms is their unique opportunity to know the surrounding environment from data input, even without assistance [33–37]. As such applying machine learning algorithm is preferable than traditional models [38–40]. A machine learning models trained on labelled data sets learn and grow more accurately over time. An algorithm would be trained with the baseline values even using labelled by humans, and the machine would learn to recognise patterns or something else on its own. Today, the most common type is supervised machine learning. The study employed four machine learning algorithms for comparison and generalisation. In the final model heart disease type, rheumatic disease type, NYHA class, age, weight, SPO2, INR, ejection fraction, duration of stay after surgery, hemoglobin level, creatinine value, waiting time for surgery variables were considered. After applying feature selection and machine learning algorithm comparison, we select the most important predictors for time to death prediction. Python jupyter notebook was implemented to perform machine learning survival modelling.

In this survival analysis the study implements different types of machine learning algorithms such as random survival forest (RSF), survival tree, support vector machine, and gradient boosted survival.

Parametric and semi parametric models often have a well-defined mathematical form with interpretable parameters, allowing for a clear understanding of how each variable contributes to the model’s predictions. This interpretability can be crucial in domains were explaining and understanding the underlying relationship between variables is essential. As such the study also implements parametric survival analysis for detail interpretations to deal the impact of each predictor on the outcome.

### Semi parametric (cox) survival

Survival analysis investigates and models the time it takes for events to occur. Survival analysis is mostly associated with death-related events, but the scope of survival analysis is much broader. The distribution of survival times is the focus of survival analysis [41]. Let T stand in for survival time. The instantaneous risk of death at time t, assuming survival to that point, can be expressed as follows: ${h}_{i}\left(t\right)=\text{exp}\left(\alpha +{\beta }_{1}{x}_{i1}+{\beta }_{2}{x}_{i2}+\cdots +{\beta }_{k}{x}_{ik}\right)$

### Parametric survival

Most common Parametric survival analysis; Exponential, Gompertz, Generalized gamma, Weibull, log normal and log logistic were considered for model fit. The study compares the most fitted model for the data.

### Model comparison

**Fig. 4:**
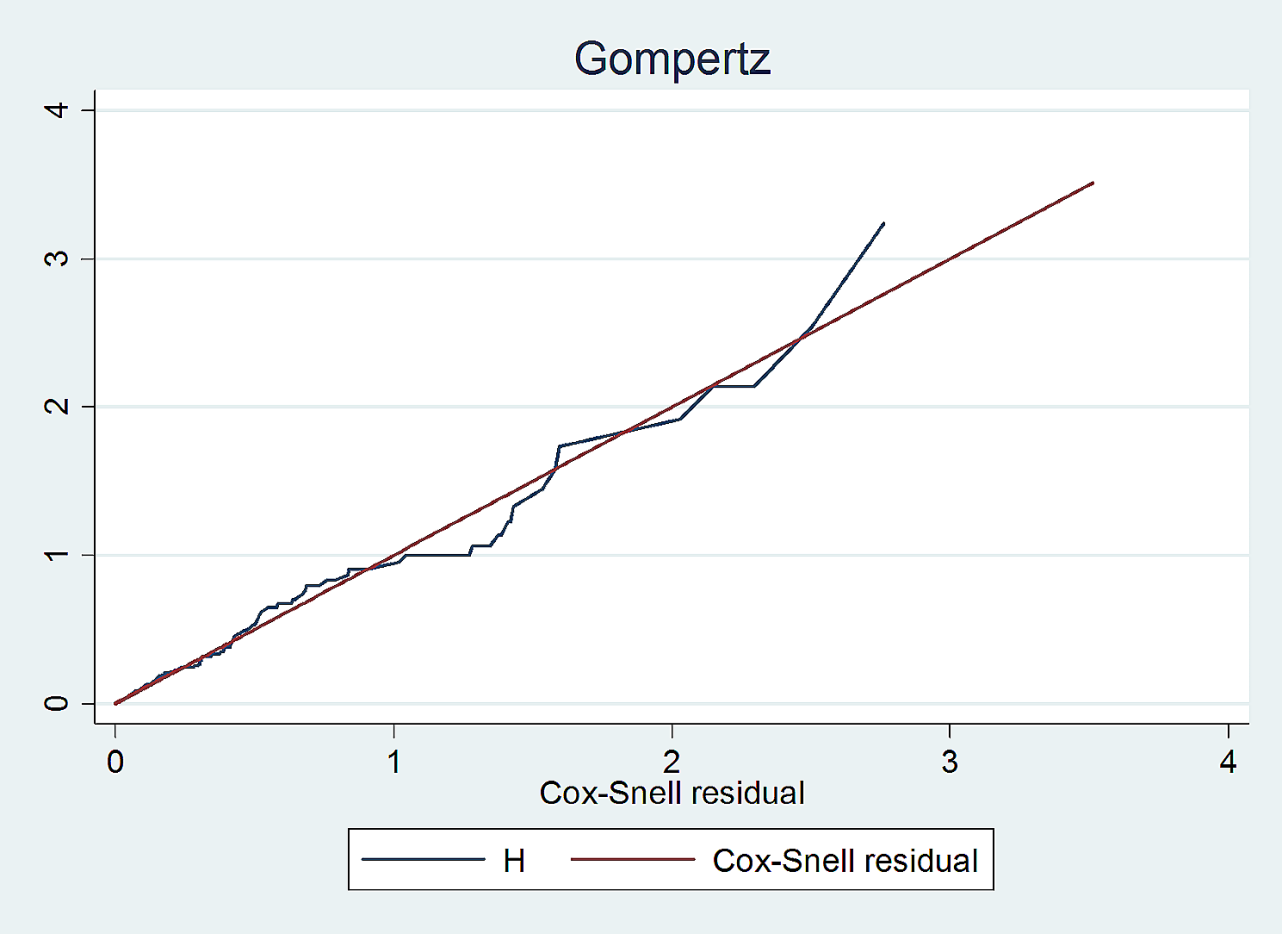
Cox Snell model fit

Parametric and semi parametric model comparison was conducted using AIC and BIC such that the one with smallest AIC and BIC was considered as the best fit. Based on model fit, the model with the smallest AIC is goes to Gompertz over other semiparametric and parametric survival models. As such the model fitted with Gompertz survival analysis (Table 1). Comparing the jagged line with the reference line, we observe that the Gompertz survival model fits the data (Fig. 4).

**Table 1:** Model comparison

Distribution type	Log likelihood (model)	AIC	BIC
Exponential	-371.9611	773.9222	853.8192
Gompertz	-366.5153	765.0306	850.2541
Loglogistic	-372.3034	776.6067	861.8302
Generalize Gamma	-369.0494	772.0989	862.6488
lognormal	-381.3373	794.6745	879.898
Weibull	-369.0702	770.1404	855.3639
Cox regression	-654.6218	1337.244	1411.814

## Result

In this study, 1520 participants were included with an average age of 24 years and an average weight of 42 kg, with average baseline systolic and diastolic blood pressure of 107 and 66, respectively. The mean oxygen saturated value was also 95%. The average value of the post-surgery ejection fraction was 59%. The Average waiting time in the cardiac center after surgery was seven days. T-test used to compare average values. There was a significant mean difference between average censored and average death for age, weigh, SPO2, INR, cardiac center duration after surgery, hemoglobin and creatine value predictors (Table 2).

**Table 2:** Post-cardiac surgery patients’ descriptive analysis for continuous variables mean comparison through t-test

Variable	Definition	Overall mean	Outcome variable		Standard deviation	T-test	P value
			Average Censored	Average Death			
Age	Baseline Age of cardiac patient	24.44	23.52003	34.93443	18.65899	-6.5693	< 0.001
Weight in KG	Baseline Weight of cardiac patient	42.48	41.65737	51.86885	22.3574	-4.8743	< 0.001
Systolic Blood Pressure	Baseline Systolic Blood Pressure	107.09	106.9979	108.2049	13.65917	-0.9361	0.349
Diastolic blood pressure	Baseline Diastolic Blood Pressure	66.88	66.82475	67.57377	10.92499	-0.7261	0.468
Pulse rate	Baseline Pulse rate	84.48	84.47568	84.46721	19.11724	0.0047	0.996
SPO2	Baseline Oxygen saturation	94.73	95.07797	90.72951	3.949677	12.2183	< 0.001
INR	International normalized ratio	2.62	2.644356	2.441148	0.7456696	2.8937	0.004
Ejection fraction	Post-surgery Ejection Fraction	59.05	59.32797	55.81148	6.774005	5.5526	< 0.001
ICU stay	Post-surgery Intensive care unit stay	1.91	1.918455	1.786885	1.085273	1.2845	0.199
Cardiac center duration	Post Length of Cardiac Center Stay in days	7.46	7.561516	6.319672	4.721736	2.7922	0.005
Waiting time	Time to surgery	480.55	483.8208	443.0861	600.3805	0.7186	0.473
Hemoglobin		12.33	12.38736	11.7042	1.68298	4.3249	< 0.001
Creatinine		0.70	0.6805579	0.9468033	0.2774684	-10.5253	< 0.001

Of the total 1520 patients, 122 (8%) patients died. From all patients who had cardiac surgery, Female (842), patients from rural area (692), Heart disease type valvular (874), congenital (593), other (53), patients with family history (145), patients with heart failure status(20), patients with smoking habit (4), Cardiac rhythm normal sinus rhythm (49), number of surgery more than one (25), Joint heart case Combination(177). From chi-square test of association, there was a significant association between outcome variable and heart disease type, rheumatic disease status, family history, NYHA class, and heart failure status (see Table 3).

**Table 3:** Frequency and chi-square test of association analysis of post-cardiac patients in Ethiopia

Predictors/categories		Number of patients	Average time of outcome variable		Chi-square value	P value
			Censored	Death		
Gender	Female	842	780 (92.64)	62 (7.36)	1.1237	0.289
	Male	678	618(91.15)	60(8.85)		
Residence	Rural	692	640 (92.49)	52(7.51)	0.4509	0.502
	Urban	828	758(91.55)	70(8.45)		
Heart disease type	Congenital	593	575 (96.96)	18 (3.04)	39.7335	< 0.001
	Other	53	42 (79.25)	11(20.75)		
	Valve Disease	874	781 (89.36)	93(10.64)		
Rheumatic disease status	Non-Rheumatic	387	372 (96.12)	15(3.88)	12.1147	0.001
	Rheumatic	1133	1,026 (90.56)	107(9.44)		
Family History	No	1375	1,273(92.58)	102(7.42)	7.2210	0.007
	Yes	145	125 (86.21)	20(13.79)		
NYHA class	II	569	543 (95.43)	26(4.57)	15.0766	0.001
	III	857	769 (89.73)	88(10.27)		
	IV	94	86 (91.49)	8(8.51)		
Baseline diabetes status	No	1506	1,385 (91.97)	121(8.03)	***	***
	Yes	14	13 (92.86)	1(7.14)		
Pulmonary hypertension status	No	1462	1,347(92.13)	115(7.87)	1.3350	0.248
	Yes	58	51 (87.93)	7(12.07)		
Stroke status	No	1516	1,397 (92.15)	119(7.85)	***	***
	Yes	4	1(25)	3(75)		
Heart Failure Status	No	1500	1,391(92.73)	109(7.27)	89.1151	< 0.001
	Yes	20	7(35)	13(65)		
Smoking status	No	1516	1,397(92.15)	119(7.85)	***	***
	Yes	4	1(25)	3(75)		
Number of surgeries	More than one	25	24 (96)	1(4)	***	***
	One	1495	1,374 (91.91)	121(8.09)		
Joint heart case	Combination	177	161(90.96)	16(9.04)	0.2786	0.598
	Single	1343	1,237(92.11)	106(7.89)		
Cardiac rhythm	Atrial fibrillation	1471	1,351(91.84)	120(8.16)	***	
	Normal sinus rhythm	49	47 (95.92)	2(4.08)		

*Note* *** shows lack of the assumptions of the chi-square test of association

The log-rank test showed a significant association between survival outcome status (Death, Alive) and heart disease type, rheumatic disease status, and NYHA class (See Table 4).

**Table 4:** Survival experience comparison of post-cardiac patients in Ethiopia

Predictors/categories		Restricted mean	Pearson Chi-Square	P value
Heart disease type	Congenital	15.25	46.92	<0.001
	Other	5.11		
	Valve Disease	11.22		
Rheumatic disease status	Non-Rheumatic	14.88	7.02	0.008
	Rheumatic	11.93		
NYHA class	II	13.18	17.35	<0.001
	III	12.33		
	IV	11.16		

The mean cross-validation score represents the average performance of the model across multiple cross-validation folds, while the model score is the performance on a specific test set. The mean cross-validation score provides an estimate of how well the model is expected to generalize to new, unseen data. It is computed by averaging the performance scores obtained from each fold of the cross-validation process. From a 10-fold cross validation and model test result, the study observe that the best survival model is random survival forest as compared to other algorithms such as Support Vector Machine, Gradient Boosted and survival tree. In classification tasks, a score of 0.89 could indicate a high level of correct predictions, where the model is accurately classifying 89% of the instances. The mean cross-validation score represents the average performance across all the folds. A score of 0.83 indicates that, on average, the model correctly predicts the outcome or class label for approximately 83% of the instances in the dataset. As such the interpretation is performed through random survival forest (see Table 5).

**Table 5:** Table average mean cross validation and model score

Machine learning survival Model	Mean cross validation	Model score
Support Vector Machine	0.79	0.85
Gradient Boosted	0.80	0.86
Survival Tree	0.70	0.76
Random Survival Forest	0.83	0.89

**Fig. 5:**
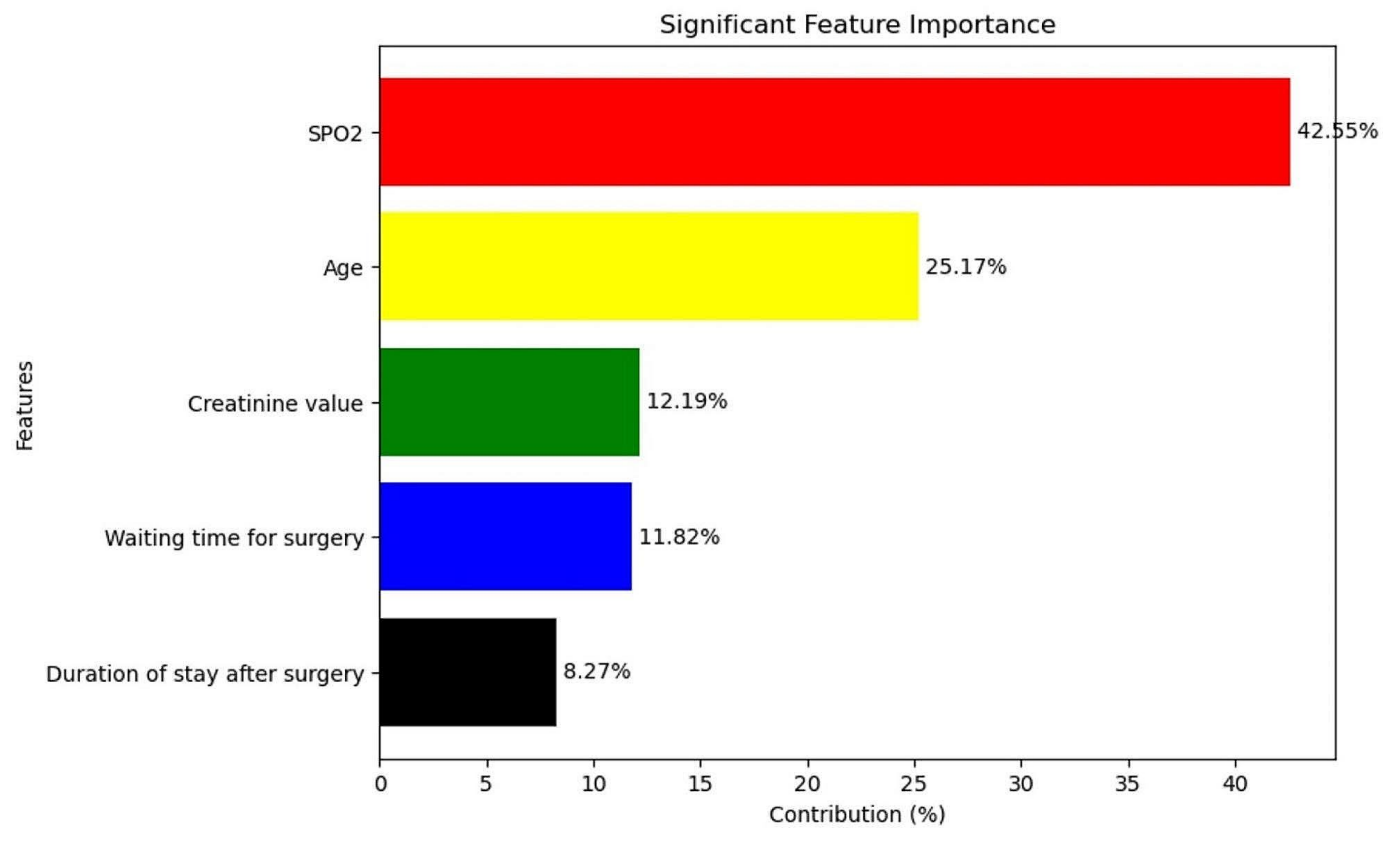
Random survival forest variable importance

From random forest survival, the graph highlights the significance of various predictor variables in the selected model. It emphasizes the importance of certain features, namely SPO2, Age, time to surgery waiting time, length of health facility leave date, and creatinine value. These variables have a significant impact on the model’s performance and play a crucial role in predicting the time to event outcome. Specifically, SPO2 contributes approximately 42.55% to the model, Age contributes around 25.17%, time to surgery waiting time contributes approximately 11.82%, length of health facility leave date contributes around 8.27%, and creatinine value contributes approximately 12.19% (Fig. 5).

From the Gompertz regression, of saturated oxygen, age, ejection fraction, length of cardiac center leave day, creatine value, and waiting time to surgery were statistically significant predictors at a 95% confidence interval.

For cardiac patients under post-surgery treatment, as age increased by one year, the mortality hazard increased by 69.2%. ((HR = 1.692, 95% CI: 1.306–2.192, *p* < 0.001). With a single rise of saturated oxygen, the hazard of mortality was reduced by 57.5% (HR = 0.425, 95% CI: 0.359–0.503, *P* < 0.001). Regarding ejection fraction, for a single increase of ejection fraction, the mortality hazard decreased by 25.3% (HR = 0.747, 95% CI: 0.629–0.887, *P* = 0.001). Regarding cardiac center duration, for a single day increase of leave day in a cardiac patient, the mortality hazard was decreased by 38.5% (HR = 0.615, 95% CI: 0.497–0.762, *P* < 0.001). About hemoglobin value, for a single unit increase in hemoglobin level, hazard of mortality was decreased by 13.7% (HR = 0.863, 95% CI: 0.741–1.005, *P* = 0.057). As a unit increase of creatinine value for cardiac patients, the hazard of mortality increased by 39% (HR = 1.364, 95% CI: 1.165–1.597, *P* < 0.001). Another most important predictor was waiting time, as a day prolonged time to surgery or waiting time for cardiac surgery, the mortality hazard was increased by 59.2% (HR = 1.592, 95% CI: 1.327–1.911, *P* < 0.001) given the other conditions constant (Table 6).

**Table 6:** Gompertz post cardiac patients’ survival multivariable result in Ethiopia

predictors	HR	P value	[95% Conf. Interval]	
			lower	upper
Heart Disease Type Other	0.568	0.330	0.182	1.773
Heart Disease Type Valvular	1.215	0.636	0.543	2.716
Heart Disease Type Congenital (Ref)				
Rheumatic disease Status (Rheumatic type)	1.389	0.383	0.664	2.907
Rheumatic Disease Status (Non-romantic) (Ref)				
NYHA Class III	1.352	0.229	0.827	2.212
NYHA Class IV	0.598	0.263	0.243	1.472
NYHA Class II (ref)				
Age	1.692	<0.001	1.306	2.192
Weight	0.831	0.243	0.610	1.133
Saturated oxygen	0.425	< 0.001	0.359	0.503
INR	0.926	0.349	0.787	1.088
Ejection Fraction	0.747	0.001	0.629	0.887
Length of cardiac center leave Day	0.615	< 0.001	0.497	0.762
Hemoglobin Value	0.863	0.057	0.741	1.005
Creatine value	1.364	< 0.001	1.165	1.597
Waiting time to surgery	1.592	< 0.001	1.327	1.911

## Discussion

The death prevalence of cardiac patients after surgery was 122(8%). The finding is similar to the previously conducted research, which is more or less similar to that found in most other studies [42–45]. After cardiac surgery, the significant predictors for heart patients were creatine value, length of post-cardiac surgery stay in the cardiac center, ejection fraction, saturated oxygen (SPO2), time to surgery, and age. The study was designed to identify the responsible predictors of post cardiac surgery mortality.

Age is one of the significant predictor variables for heart disease death, and this result is aligned with other previously conducted studies [46–48], consistent with the investigation. It is because when the patients age increases the possibilities of complication and other comorbidity also increases. And the responsiveness of the treatments also decreases when age increases. Physiological aging of the heart as a major causative predictor in the manifestation and onset of cardiac in aging due to increased inflammation and oxidative stress [49].

Other studies [50–52] also showed that creatinine is essential in predicting mortality after heart surgery. The study demonstrated the feasibility of using serum creatinine as an outcome indicator in post-cardiac surgery [53]. The critical role of creatinine as a strong predictor has been incorporated in the different mortality risk scores currently used for cardiac surgery patients [54–56]. Creatinine is a chemical waste product. This test is done to see how well your kidneys work. Creatinine is removed from the body entirely by the kidneys. Elevated creatinine level signifies impaired kidney function or kidney disease. As the kidneys become impaired for any reason, the creatinine level in the blood will rise due to poor clearance of creatinine by the kidneys. Abnormally high levels of creatinine thus warn of possible malfunction or failure of the kidneys. When the heart is no longer pumping efficiently it becomes congested with blood, causing pressure to build up in the main vein connected to the kidneys and leading to congestion of blood in the kidneys, too this might facilitate mortality of cardiac patients.

Saturated oxygen was the main predictor for predicting death [57]. Reduced tissue oxygen saturation may be associated with poor postoperative outcomes in cardiac surgery patients [58]. Management and understanding of patient care depend on oxygen saturation. The level of oxygen within the body is tightly regulated as hypoxemia can have a wide variety of acute adverse effects on different organ systems depending on the degree of hypoxia. These include the brain, heart, and kidneys. The blood can’t deliver enough oxygen to your organs and tissues if it has low oxygen levels (hypoxia). If it persists for a long time, it can damage your heart and brain. When hypoxemia occurs acutely, it can be fatal. Such a situation might increase the risk of mortality.

Left Ventricular Ejection Fraction (LVEF) indicates the efficiency of the ventricle and is regarded as an optimal marker of LV function. Left ventricular ejection fraction has been considered among the strongest predictors of clinical outcomes after cardiac surgery [59, 60]. Left Ventricular Ejection Fraction had a significant association with mortality [61]. The ejection fraction low value was the main death predictor, which agrees with [50, 62–65]. Variables that have often been shown to predict mortality ejection fraction were predictive in this population [47]. Ejection fraction is an indicator of heart strength. It measures the amount of oxygen-rich blood pumped out to the body with each heartbeat. A low ejection fraction is an indicator that the heart can’t plumb enough blood and this further leads to its failure. A low ejection fraction number can be an indicator of heart failure and may not have symptoms at first but can lead to a variety of symptoms, like shortness of breath. This might be the case to drive mortality.

Hemoglobin value was related to worse outcomes. This value is probably related to the patient’s previous comorbidities-related issue, contributing to a worse death outcome in agreement with a study [45]. Lower hemoglobin value has been identified as a predictor of poor short- and long-term outcomes in a nonoperative setting in the general and elderly population [66, 67], in patients with coronary artery disease [68, 69], and in patients with congestive heart failure [70]. Recently, several studies addressed pre-operative Lower hemoglobin as a predictor of poor short-term outcomes after cardiac surgery [71–75]. Pre-operative lower hemoglobin values during cardiopulmonary bypass have been identified as significant risk factors for blood transfusion during cardiac surgery [76]. Lower hemoglobin value leads to anemia. The anemia itself can worsen cardiac function, both because it causes cardiac stress through tachycardia and increased stroke volume, and because it can cause reduced renal blood flow and fluid retention, adding further stress to the heart.

The study on hospital mortality found that increased length of postoperative hospital stay after cardiac surgery is associated with an increased likelihood of in-hospital mortality [77], which is inconsistent with the study; the difference may arise from the study follow-up period and study design. another reason might be since patients are from a limited economic setting, the required money for hospitality might be difficult to afford as such patients might withdraw from treatment, especially for private treatments too early which increases the risk of mortality.

Waiting time was highly related and the most significant predictor of cardiac patients’ post-surgery mortality. However, some papers stated that the surgery outcome was not related to the waiting time [78]. Another study stated that Prolonged waiting was not associated with worse surgical outcomes [79]. Those studies contradicted the study just due to the study area with a lower waiting time unlike to developing country Ethiopia. In Ethiopia, the number of cardiac centers was too few as compared to the case. As of only two fully active cardiac centers for more than 120 million population. Another difference might be the coverage of cardiac disease type.

## Conclusion

In conclusion, lower saturated oxygen, prolonged waiting time, aged, lower ejection fraction, short period cardiac center duration after surgery, and lower creatine values were responsible for time-to-death outcomes. Special attention is required for surgery patients under follow-up with those parameters. There are only two fully active cardiac centers in Ethiopia for more than 120 million people, at the end, the study highly and urgently recommended to increase the number of cardiac centers in Ethiopia. The study recommends that clinicians, the Ministry of Health, policymakers, and the general public raise awareness and develop policies for cardiac heart disease patients to facilitate better management and save lives.

### Electronic supplementary material

Below is the link to the electronic supplementary material.

Supplementary Material 1

## Data Availability

The data used in the current study is available under the corresponding author and can be attached on request.

## Abbreviations

NYHA: New York Health Association
SPO2: Oxygen Saturated
INR: International Normalised Ratio
CVD: Cardiovascular
LMICs: low and middle-income countries
