# Supplementary material for: Post-surgery survival and associated factors for cardiac patients in Ethiopia: applications of machine learning, semi-parametric and parametric modelling
Source: BMC Med Inform Decis Mak. 2024 Mar 29;24:91. doi: 10.1186/s12911-024-02480-9 (PMC10979627; doi:10.1186/s12911-024-02480-9)
Supplement: Supplementary file 1 — Supplementary Material 1 [file 12911_2024_2480_MOESM1_ESM.docx]

Supplementary file 1: Questionnaire developed to assess cardiac surgery patients' follow-up

| Variable | category | remark |
| --- | --- | --- |
| Sex | 1. Male 2. Female |  |
| Age | ------------- |  |
| Residence | 1. Urban 2. Rural |  |
| Weight in KG | ------------- |  |
| Family history of cardiac heart disease | 1. Yes 2. No |  |
| Smoking status | 1. Yes 2. No |  |
| Baseline diabetes status | 1. Yes 2. No |  |
| Baseline creatinine value | ----------------- |  |
| Patients’ Admission Date | ___/_____/___________ | DD/MM/YY |
| Patient's surgery date | ___/_____/___________ | DD/MM/YY |
| Patient’s last visit date | ___/_____/___________ | DD/MM/YY |
| Patients Death certificate date (if death) | ___/_____/___________ | DD/MM/YY |
| ICU stay in days | ----------- |  |
| Systolic blood pressure in number | ----------------- |  |
| Diastolic blood pressure in number | ------------------- |  |
| INR in number | -------------------- |  |
| Pulse rate in number | ---------------- |  |
| Rheumatic heart disease type | 1. Rheumatic heart disease 2. Non-Rheumatic heart disease) |  |
| Cardiac center leaves Day after surgery | ___/_____/___________ | DD/MM/YY |
| Hemoglobin Value in number | -------------------- |  |
| Pulmonary hypertension status | 1. Yeas 2. No |  |
| NYHA class | 1. I 2. II 3. III 4. IV |  |
| Left ventricular ejection fraction (LVEF) in percent | ------------------------------------ |  |
| Stroke status | 1. Yes 2. No |  |
| Heart Failure Status | 1. Yes 2. No |  |
| Number of surgeries | 1. one 2. Above one |  |
| Joint heart disease case | 1. Single 2. Combination |  |
| Cardiac rhythm | 1. Atrial fibrillation 2. Normal sinus rhythm |  |
| Survival outcome | 1. Alive 2. censored 3. Death |  |
